# Near-Peer Teaching in Radiation Oncology: a Proof of Principle Study for Learning Treatment Planning

**DOI:** 10.1007/s13187-022-02150-2

**Published:** 2022-05-03

**Authors:** Gerard M. Walls, Rachel Ellis, Sophie Lynch, Margaret A. Flynn, Gemma McCann, Lucy J. Jellett, Claire Harrison

**Affiliations:** 1 Cancer Centre Belfast City Hospital, Belfast Health & Social Care Trust, Lisburn Road, Belfast, BT9 7AB Northern Ireland; 2 Patrick G Johnston Centre for Cancer Research, Queen’s University Belfast, Jubilee Road, Belfast, BT9 7AE Northern Ireland; 3 North West Cancer Centre, Western Health & Social Care Trust, Glenshane Road, Derry, BT47 6SB Northern Ireland

**Keywords:** Near-peer teaching, Treatment planning, Radiation oncology, Interprofessional, Quality improvement

## Abstract

Radiotherapy techniques are expanding in range and complexity; therefore, protecting learning environments where residents nurture treatment planning skills is critical. The evidence base for ‘near-peer’ teaching (NPT), where professionals at a similar career stage assist in each other’s learning, is growing in hospital-based disciplines, but has not been reported in radiation oncology. The feasibility of a resident-led teaching programme for developing treatment planning skills was investigated herein with quality improvement (QI) methodology. Following consultation with attendings (*n* = 10) and all residents (*n* = 17) at the two cancer centres in the region, a regular NPT session focused on planning skills was initiated at the largest centre, with video-linking to the second centre. Tutorials were case-based and pitched at the level of qualifying examinations. Plan–Do–Study–Act (PDSA) cycles were designed based on primary and secondary improvement drivers derived by group consensus among residents, with tutorials adopted accordingly. Participation, content, and satisfaction were monitored for 20 months. Six PDSA cycles reformed the tutorial format, leading to logistical and pedagogical benefits including interprofessional contributions and enhanced interactivity. Tutorials occurred on 85% prescribed occasions (*n* = 45) during the subsequent 18-month follow-up, with 25 distinct tumour sites featured. Resident participation and satisfaction increased, independent of resident seniority. Tutorials were paused for the first 2 months of the SARS-CoV-2 pandemic only. A high-quality and cost-effective regional, trainee-led teaching programme on treatment planning was feasible and cost-effective in this study.

**Supplementary Information:**

The online version contains supplementary material available at 10.1007/s13187-022-02150-2.

## Introduction

Prior to independent practice, radiation oncologists must accrue a theoretical understanding and sufficient practical experience of the blend of contemporary and conventional paradigms offered by modern centres. Rising cancer incidence and expanding indications pose a challenge for programme directors who strive to balance training responsibilities and safe service delivery. There is recognition internationally that residents struggle to attain planning competencies as a result [1–4], and dedicated academic groups seek to identify novel solutions [5]. Near-peer teaching (NPT) is one medical education intervention showing promise in other hospital disciplines [6]. Defined as “people from similar social groupings helping each other to learn, and learning themselves by teaching”, NPT pivots on the concepts of social and cognitive congruence. Cognitive congruence suggests that a teacher with similar knowledge to the learner may sometimes be a more effective teacher than an expert [7, 8]. The social congruence of a teacher being in a similar role may similarly instil a more relaxed and less intimidating learning environment. Hypothesizing that the accumulation of planning skills may be amenable to NPT, a resident-led programme was instigated and assessed in our region employing a formal quality improvement (QI) approach.

## Methods

### Study Design

A root cause analysis for suboptimal resident experiences of treatment planning was performed during a meeting of residents in February 2019, summarized in the affinity chart (Table 1). Primary and secondary drivers for the NPT programme were also derived during this brainstorming meeting [9] (Supplementary Index, Fig. A). Self-nominated residents leading sessions focussed on the tumour sites they most recently completed was the original programme outline decided by consensus, with sessions on alternate weeks (or weekly approaching qualifying examinations).

**Table 1:** Affinity diagram

Why do residents receive limited exposure to treatment planning?		
Resident factors	Attending factors	Departmental factors
• Limited available time • ‘On call’ clinical duties • Low confidence • Unfamiliar terminology • Inadequate physics knowledge	• Limited available time • Reliance on ad hoc working • Relationship with resident • Enthusiasm for teaching	• Limited induction • Lack of radiology teaching • Restricted space/monitors • Lack of software licences • Exam-based permissions

Arranging dosimetrist input was encouraged for complex technical aspects where possible. The elected resident coordinator’s term was set at 1 year. The programme commenced (i.e. the intervention) in March 2019 with a prospective plan to undertake a Plan–Do–Study–Act (PDSA) cycle each month for 6 months in order to iteratively advance the programme, based on resident group feedback. Participation, content, and satisfaction were prospectively recorded over a 20-month period **(**Table 2**)** using a bespoke survey. The survey text was distributed and returned in hospital emails between RO residents and the authors (GW or RE) (Supplementary Index, Questionnaire). Ethical approval was waivered by the hospital organization as quality improvement projects are exempt.

**Table 2:** Participation and satisfaction levels according organized by study period (*survey data from *n* = 12 trainees)

		Baseline	Early follow-up	Intermediate follow-up	Late follow-up
Frequency of meetings (% of total availability)		25*	72	78	64
Resident attendance (% of 15 available residents)		25*	43	42	50
Attending oversight (% of all tutorials)		25*	46	29	55
		Baseline (*n* = 13)	Early follow-up (*n* = 17)	Intermediate follow-up (*n* = 13)	Late follow-up (*n* = 12)
Benefit of attendings’ input, %	Yes	100	X	100	100
	No	0		0	0
Session interruption, %	All	0	X	0	0
	Most	8		23	8
	Occasional	66		69	75
	None	25		8	17
Improved ‘on the job’ learning, %	Yes	83	X	100	100
	No	17		0	0
Usefulness for junior residents, %	Crucial	38	X	23	50
	Useful	62		77	50
	Not useful	0		0	0
Usefulness for senior residents, %	Crucial	46	X	85	100
	Useful	38		15	0
	Not useful	16		0	0

### Context

The region has approximately 17 residents, some of whom fulfil service provision roles predominantly. Two centres provide tertiary care for all tumour types in the region, with > 5000 new cases referred annually. Advanced techniques available include arc therapy, breath-hold techniques, 4-dimensional planning, and stereotactic treatment. A half-day of small group teaching by attendings in oncology and related specialties occurs monthly, and residents present cases at a weekly ‘Grand Rounds’ meeting attended by the entire faculty. The larger centre houses a dedicated oncology library, and residents are supported to attend courses before qualifying examinations.

### Statistical Analysis

Descriptive statistics were used to tabulate results and graphs were produced using Prism 8.0 (GraphPad Software, San Diego, CA, USA). Tests for statistical significance were not undertaken given the small population size involved.

## Results

### Participation

A total of 45 tutorials (85%) took place from a possible total of 53 weeks during the 20-month study period (excluding weeks of examinations, Christmas, Easter, and the first pandemic surge). Twenty-five different tumour sites were covered, with more common tumour sites being repeated. Mean resident participation was 67% (*n* = 8, range 4–12) adjusting for actual trainee availability (typically 5 residents are unavailable due to ‘on call’ commitments or planned leave). All residents presented on ≥ 1 occasion (range 1–5). A medical dosimetrist participated in 27 sessions (60%). An attending was available for 20 sessions (44%), and radiation therapist input was utilized for 5 sessions (11%).

### Satisfaction

**Fig. 1:**
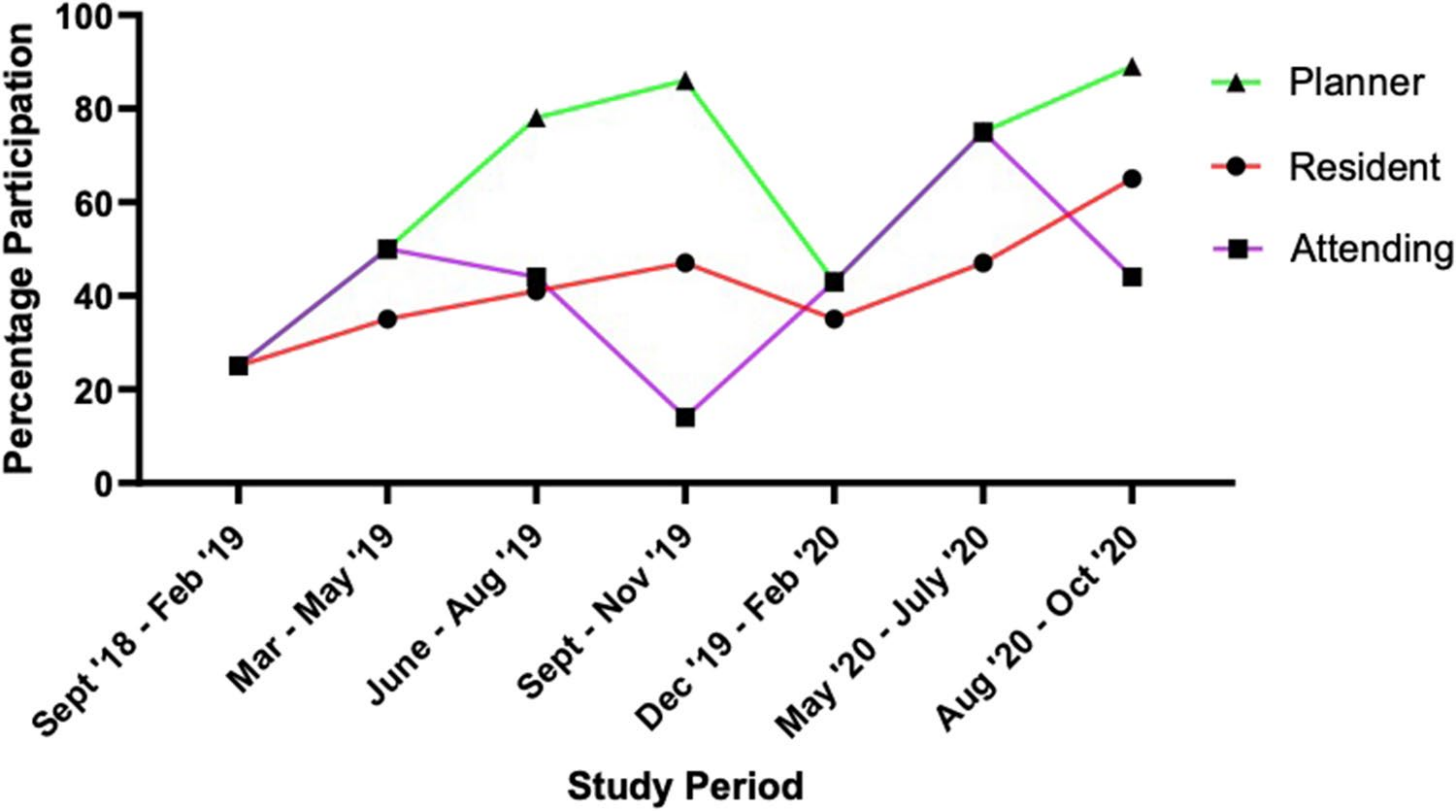
Run chart for the project (by quarter)

The mean response rate for satisfaction ratings was 74% (range 71–76). Ratings were not collected at the early time point as the interval between some of the more recent of the sequentially introduced interventions was short. Improvement in ‘on the job’ learning from the sessions increased from 83 to 100%. All respondents found the programme to be beneficial. The proportion of respondents indicating that senior residents and junior residents rating the course as ‘crucial’ from the tutorials increased from 46 to 100%, and 38 to 50% respectively. The value of attending input was rated as ‘crucial’ by all residents at all time points. The frequency of interruptions for non-emergency clinical scenarios was ‘occasional’ for 66% of residents at baseline and this was stable throughout the study. Monthly trends are depicted in a run chart (Fig. 1) and displayed by intervention period in the Supplementary Index, Table A. There were no associated financial costs as all necessary resources were available.

### Plan–Do–Study–Act Cycles

#### Cycle 1—Venue Suitability

Following two unsuccessful attempts, the third candidate venue trialled by the resident group met their desired criteria, which included intimate size, large display, video-link capacity, reliable availability, and privacy from hospital thoroughfares.

#### Cycle 2—Protected Time

The programme director decreed 1 h of protection from clinical interruptions to maximize the benefit of tutorials. A non-training resident responded to any such calls initially until his contract ended, from which point the resident on referrals assumed this duty.

#### Cycle 3—Regular Interprofessional Input

Two medical dosimetrists committed to routinely contributing to tutorials. Radiation therapists from the pre-treatment department (scanning and mould room) contributed to relevant sessions, e.g. skin cancer.

#### Cycle 4—Attending Oversight

Access to a sub-specialized attending during tutorials for resolving complex nuances was highlighted by residents. To maximize engagement from attendings, an online survey was first conducted to elicit attending views and a high level of enthusiasm was evident (data not shown).

#### Cycle 5—Interactive Format

To engender both depth of discussion and an informal atmosphere, presenting residents were permitted to teach on tumour sites of their choice. Medical dosimetrists upgraded from paper-based to digital formats for plan evaluation exercises, to improve participant experience at the second centre. Residents were exempt from presenting during their first 6 months to promote a positive ethos.

#### Cycle 6—Case Repository

Given that five residents are typically unavailable due to ‘on call’ commitments or planned leave, making tutorial materials available afterwards was agreed, also serving to generate a bank of cases for future examination candidates.

## Discussion

The balance between service provision and training opportunities is increasingly challenging to achieve due to increasing caseloads [10, 11]. Although much of the knowledge and skill needed for treatment planning is learnt ‘on the job’ in an apprenticeship-type model [12, 13], it is prudent that all of the educational resource available in the workplace is harnessed. Anecdotally, many radiation oncologists recall valuable informal learning experiences with fellow residents during their training. Herein, we demonstrated the feasibility of regular, trainee-led, small group teaching focussed on treatment planning skills.

Sessions were well attended by residents, medical dosimetrists, and visiting attendings, and these trends improved over time. A broad spectrum of topics was covered and satisfaction levels were persistently high. Excusing an unavoidable hiatus at the onset of the SARS-CoV-2 pandemic, the programme proved to be sustainable, enduring subsequent pandemic surges due to its previously established digitally enabled format. Importantly, this feature also permitted self-isolating or re-deployed residents to participate. Furthermore, as hospital pressures precluded attending-led teaching and Grand Rounds, the NPT sessions were of profound significance for residents during the pandemic.

In addition to a novel learning resource for residents, opportunities for teaching practice and presenting experience were generated. Furthermore, collaboration with dosimetrists and radiation therapists led to the forging of new interprofessional relationships, previously reported to be highly beneficial [14–17].

Not all of the parameters improved steadily however. Dosimetrist and resident participation appeared to fall transiently in December 2019–February 2020, although this is likely a reflection of service disruption around the holiday season. Interruptions remained an issue despite targeted interventions, most likely due to resistance within the workplace culture as opposed to individuals, both in terms of the attitudes of the resident’s readiness to be contacted and the tendency of colleagues to make contact during protected teaching [12].

PDSA cycles, a central mechanism within QI framework (outlined in Supplementary Index, Fig. B [18]), were central to the incremental enhancement of the NPT programme. Audit methodology has been embedded in radiation therapy departments historically [19], and although QI processes rely on measuring compliance with a standard also, its continuous nature and adaptability for local, bespoke scenarios means QI can be preferable. Training in QI is now incorporated in radiation oncology curricula and is regarded highly by programme directors [20]. The adjustments resulting from PDSA cycles aimed to optimize the session for residents’ educational needs culminated in the production of ‘ground rules’ (Supplementary Index, Fig. C).

This study had a number of weaknesses. The common criticism of NPT being less applicable to complex topics was mitigated in this project the presence of an attending specializing in the tumour site. In this way, the onus remains on the presenting resident but expert knowledge was available, and this format was rated highly consistently. Whilst semi-quantitative satisfaction data were collected, more objective data from ‘harder’ endpoints such as pre- and post-session assessments was not available, and as such, only the first level of the Kirkpatrick Four Level Evaluation Model [21] was engaged in this study. Furthermore, the direct effect of the NPT on overall clinical aptitude would be difficult to elicit as several concurrent modifications have been made to the local training programme in recent years. Therefore, deriving the NPT tutorials’ direct impact on regional examination pass rates, for example, would be inappropriate.

It is planned that the NPT programme will undergo a further round of PDSA cycles following the easing of social distancing regulations. These will include moving to an online contouring system, to emulate the anticipated change in style of the qualifying examinations in the near future. A ‘basics of physics’ session for new residents will also be implemented. An audit of calls received is planned to examine the nature of these distractions; however, any mechanisms for lowering distractions must ultimately prioritize patient safety.

## Conclusions

A valuable, sustainable, cost-effective, and resident-led programme for developing treatment planning acumen was established through concerted resident effort and interprofessional engagement. QI framework can propagate the success of such programmes through handcrafted bespoke solutions.

## Supplementary Information

Below is the link to the electronic supplementary material.

Supplementary file1 (DOCX 67 KB)
