# Correction to: Near-Peer Teaching in Radiation Oncology: a Proof of Principle Study for Learning Treatment Planning

**DOI:** 10.1007/s13187-022-02186-4

**Published:** 2022-06-02

**Authors:** Gerard M. Walls, Rachel Ellis, Sophie Lynch, Margaret A. Flynn, Gemma McCann, Lucy J. Jellett, Claire Harrison

**Affiliations:** 1 Cancer Centre Belfast City Hospital, Belfast Health & Social Care Trust, Lisburn Road, Belfast, BT9 7AB Northern Ireland; 2 Patrick G Johnston Centre for Cancer Research, Queen’s University Belfast, Jubilee Road, Belfast, BT9 7AE Northern Ireland; 3 North West Cancer Centre, Western Health & Social Care Trust, Glenshane Road, BT47 6SB Derry, Northern Ireland

**Correction to** **:** **Journal of Cancer Education**

10.1007/s13187-022-02150-2

The original version of this article unfortunately contained an incomplete version of Fig. 1. Below is the corrected Fig. 1 complete with trend lines.
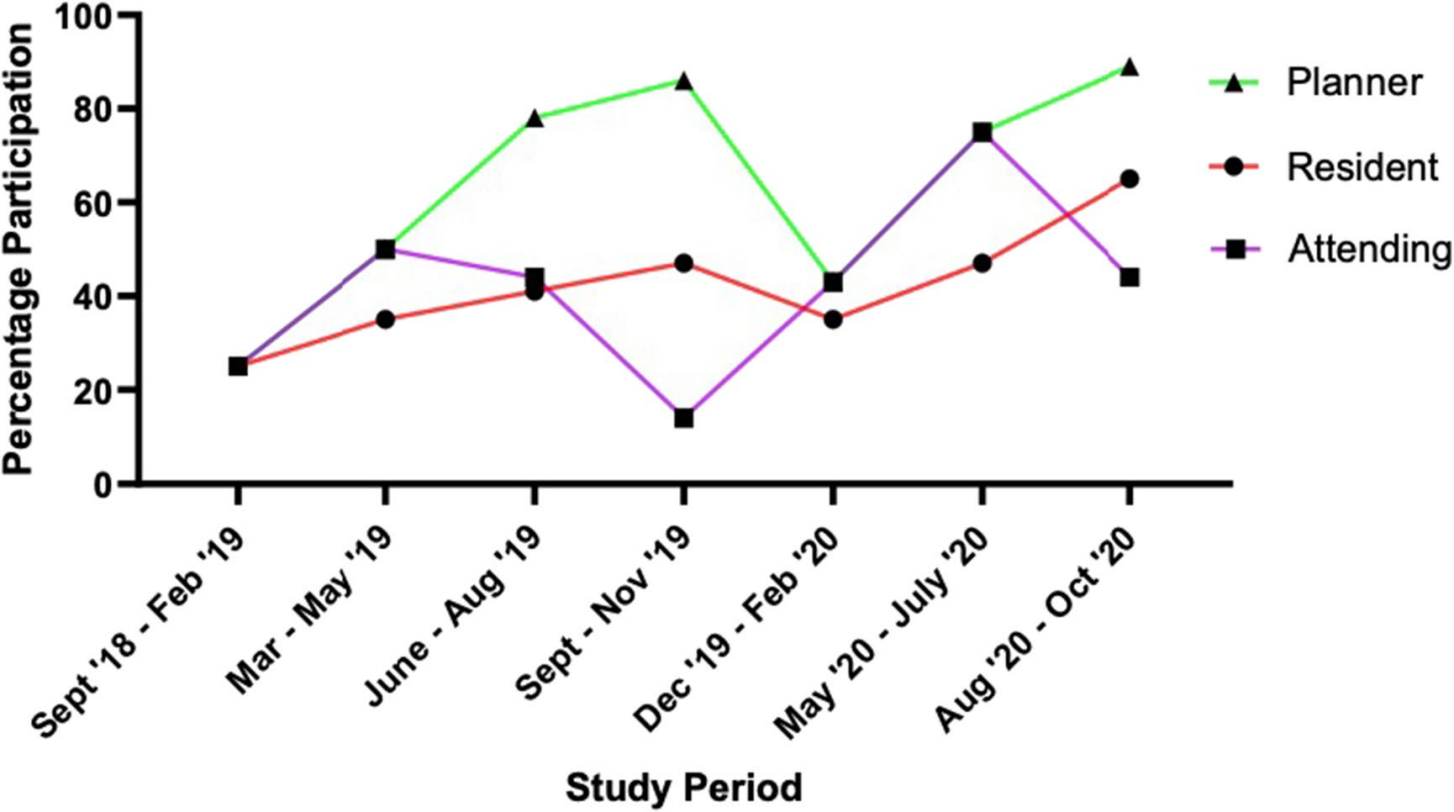


The original article has been corrected.

