# Supplementary material for: Near-Peer Teaching in Radiation Oncology: a Proof of Principle Study for Learning Treatment Planning
Source: J Cancer Educ. 2022 May 3;37(4):1245–50. doi: 10.1007/s13187-022-02150-2 (PMC9064121; doi:10.1007/s13187-022-02150-2)
Supplement: Supplementary file 1 — Supplementary file1 (DOCX 67 KB) [file 13187_2022_2150_MOESM1_ESM.docx]

**SUPPLEMENTARY INDEX**

**FIGURES**

**Figure A.** Driver diagram

**Figure B.** Examples of key steps in QI methodology

**GENERAL**

- Tutorials organized for Friday afternoons on alternate weeks
- Occurrence can increase to weekly for the quarter before examinations
- Venue criteria: size, display hardware, video-link software
- Residents are not expected to take calls during tutorials
- All residents are required to present biannually as a minimum
- New residents will not be expected to lead sessions for six months
- Room bookings with suitable video-linking will be organized in advance

**FORMAT**

- Focus is treatment planning, see table below for further detail
- Presenting residents should endeavour to restrict tutorials to ≤1 hour
- Pictures/diagrams encouraged, text-predominant slides discouraged
- Treatment field/volume exercises (paper) should be incorporated

| **Recommended** | **Not Recommended** |
| --- | --- |
| staging & anatomy | epidemiology |
| radiotherapy indications | risk factors |
| consent process | chemo (unless concurrent) |
| immobilisation & scanning | landmark trial details |
| planning technique |  |
| target volume/OAR delineation |  |
| prescription details |  |
| plan evaluation |  |

**PRESENTING RESIDENT CHECK-LIST**

1. Recruit an attending to provide oversight (4 weeks’ notice)
2. Plan session with medical dosimetrist (2 weeks’ notice)
3. Circulate images for field border exercises (48 hours’ notice)
4. Upload teaching materials to the shared folder within 1 week

**Figure C.** Agreed ‘ground rules’ for the treatment planning NPT program

**TABLES**

| **Study Period** | **Begin** | **End** | **Duration** |
| --- | --- | --- | --- |
| **Pre-Intervention** | September 2018 | February 2019 | 6 months |
| **Early post-intervention**  ***March***  ***April***  ***May***  ***June***  ***July***  ***August*** | March 2019 | August 2019 | 6 months |
|  | PDSA Cycle 1 – venue change x 2  PDSA Cycle 2 – designated contact  PDSA Cycle 3 – interdisciplinary input  PDSA Cycle 4 – attending oversight  PDSA Cycle 5 – interactive format  PDSA Cycle 6 – case repository | |  |
| **Intermediate Post-Intervention** | September 2019 | February 2020 | 6 months |
| **Onset of SARS-CoV-2 Pandemic** | March 2020 | April 2020 | 2 months |
| **Late post-intervention** | May 2020 | November 2020 | 6 months |

**Table A.** Study period definitions

**QUESTIONNAIRE**

1. **During [time period], how frequently were you interrupted for non-emergency issues during the radiation therapy treatment planning NPT sessions?**

A. All

B. Most

C. Occasional

D. None

1. **Do you think attending involvement in the radiation therapy treatment planning NPT sessions is advantageous?**

A. Yes

B. No

C. Not sure

1. **Do the radiation therapy treatment planning NPT sessions increase your rate of ‘on the job’ learning in the radiation therapy department?**
2. Yes
3. No
4. Not sure
5. **How useful do you think the radiation therapy treatment planning NPT sessions are during the first two years of training?**

A. Crucial

B. Useful

C. Not useful

1. **How useful do you think the radiation therapy treatment planning NPT sessions are during later years of training?**

A. Crucial

B. Useful

C. Not useful
